# l-Cysteine-Assisted Synthesis of Urchin-Like *γ*-MnS and Its Lithium Storage Properties

**DOI:** 10.1186/s11671-016-1664-6

**Published:** 2016-10-03

**Authors:** Dan Xu, Ranran Jiao, Yuanwei Sun, Dezhi Sun, Xianxi Zhang, Suyuan Zeng, Youying Di

**Affiliations:** Shandong Provincial Key Laboratory of Chemical Energy Storage and Novel Cell Technology, School of Chemistry and Chemical Engineering, Liaocheng University, Liaocheng, 252059 China

**Keywords:** Lithium ion battery, Urchin-like *γ*-MnS microstructures, l-cysteine, Solvothermal

## Abstract

MnS has been attracting more and more attentions in the fields of lithium ion batteries (LIBs) because of its high energy density and low voltage potential. In this paper, we present a simple method for the preparation of urchin-like *γ*-MnS microstructures using l-cysteine and MnCl_2_ · 4H_2_O as the starting materials. The urchin-like *γ*-MnS microstructures exhibit excellent cycling stability (823.4 mA h g^−1^ at a current density of 500 mA g^−1^, after 1000 cycles). And the discharge voltage is about 0.75 V, making it a good candidate for the application as the anode material in LIBs. SEM, TEM, and XRD were employed to inspect the changes of the active materials during the electrochemical process, which clearly indicate that the structural pulverization and reformation of the *γ*-MnS microstructures play important roles for the maintenance of the electrochemical performance during the charge/discharge process.

## Background

Nowadays, lithium ion batteries (LIBs) have been widely used in our daily lives, such as cellphones, laptops, electric vehicles, and other portable electrical devices [1–4]. Though graphite has been widely used in the commercial LIBs, the relatively low theoretical capacity (372 mA h g^−1^) and poor rate performance severely limits its application in various fields [5]. Thus, many efforts are devoted to developing electrode materials with high capacity, cycling stability, and low cost.

Recently, transitional metal sulfides such as cobalt sulfides [6–8], nickel sulfides [9, 10], copper sulfides [11, 12], ferric sulfides [13, 14], and manganese sulfides [15–20] have attracted much attention as an alternative for anode material in LIBs. As an important member of transitional metal sulfides, MnS is considered to be a good candidate for the next generation of anode material because of its high energy density (~616 mA h g^−1^) and low voltage potential (average discharge voltage at ~0.65 V and charge voltage at ~1.25 V) [16, 17, 20, 21]. For this reason, a great number of MnS micro-/nanostructures with different morphologies have been successfully prepared, all of which show excellent electrochemical performance when used as the anode materials in LIBs. For instance, Robinson and co-workers have successfully prepared MnS nanoparticles using the electrophoretic deposition (EDP) method, which can deliver a reversible capacity of 470 mA h g^−1^ at a current density of C/5 after 100 cycles [12]. Zhang’s group has also succeeded in the preparation of coral-like α-MnS using a facile two-step method. Because of the unique structures, the as-synthesized coral-like α-MnS exhibits excellent electrochemical performance. Under a current density of 500 mA g^−1^, the as-synthesized coral-like α-MnS can still deliver a reversible capacity of 699 mA h g^−1^ after 400 cycles [18]. Kang’s group have also reported the synthesis of MnS-C using the spray drying process, which exhibits a discharge capacity of 786 mA h g^−1^ at a current density of 500 mA g^−1^ after 100 cycles [19]. Despite these remarkable progresses, the electrochemical performance of MnS-based anode material is still far from satisfying when considering its practical use in LIBs. According to the previous reports, the relatively poor cycling stability and low discharge capacity are still the main problems that prevent them from large-scale application.

It is well known that the electrochemical performance of the electrode material is highly dependent on its morphology and crystalline texture [5, 6]. Thus, a MnS nanostructure with proper morphological design would be expected to greatly enhance the electrochemical performance of the anode material. Herein, we reported the synthesis of urchin-like *γ*-MnS via a simple solvothermal method using l-cysteine and MnCl_2_ · 4H_2_O as the raw materials. Thanks to the unique 3D structure, the as-synthesized urchin-like *γ*-MnS exhibits excellent cycling stability. The discharge capacity can still reach 823.4 mA h g^−1^ after discharging for 1000 cycles at the current density of 500 mA g^−1^. Besides the cycling stability, the as-prepared urchin-like *γ*-MnS structures also exhibit satisfying rate performance. SEM, TEM, and XRD were employed to inspect the changes of the active materials during the electrochemical process, which clearly indicate that the structural pulverization and reformation of the *γ*-MnS structures play importance roles for the maintenance of the electrochemical performance during the charge/discharge process.

## Methods

### Preparation of the Urchin-Like *γ*-MnS Nanostructures

The urchin-like *γ*-MnS nanostructures were synthesized via a facile solvothermal method. In a typical experiment, 1 mmol MnCl_2_ · 4H_2_O was firstly dissolved in the mixed solvent composing of 10 mL double-distilled water and 20 mL diethylene glycol (DEG). The mixed solution was then heated to 70 °C under constant stirring. In the next step, 2 mmol l-cysteine was added to the above solution. After heating at 70 °C for 2 h, the white turbid liquid was transferred into a 50-mL Teflon-lined stainless steel autoclave and heated to 180 °C for 18 h. After cooling down to room temperature, the ochre product was collected by centrifugation and washed three times with water and ethanol. The as-obtained product was finally dried in vacuum at 60 °C for 12 h.

### Sample Characterizations

Phase purities of the as-prepared samples were characterized using X-ray powder diffraction (XRD, Bruker D8 advanced diffractometer with Cu-Kα radiation, λ = 1.5406 Å). The sizes and morphologies of the samples were investigated using the field emission scanning electron microscopy (FESEM, Hitachi S-4800) and transmission electron microscope (TEM, Hitachi H7700, 120 kV). The high-resolution transmission electron microscopy (HRTEM) images were taken using a transmission electron microscopy (TEM, JEOL-2010) with an accelerating voltage of 200 kV. The surface information as well as chemical composition of the samples were examined using the X-ray photoelectron spectrum (XPS, ESCALAB250). The specific surface area and pore size distribution were determined by the Brunauer-Emmett-Teller (BET) nitrogen adsorption and desorption apparatus (Quantachrome autosorb IQ-C).

### Electrochemical Measurement

The working electrodes were prepared by mixing *γ*-MnS, carbon black (Super-P) and carboxyl methyl cellulose binders (CMC) with a weight ratio of 5:3:2. The slurry was coated on copper foil and then dried in vacuum at 100 °C for 12 h. And the active material on each copper foil was weighted to be ~1.46 mg cm^−2^. The CR2032 coin cells were assembled in an argon-filled glove box (Mikrouna, Super (1220/750)) with moisture and oxygen concentrations below 0.1 ppm. The electrolyte was a solution of 1 M LiPF_6_ in a mixture of ethylene carbonate (EC), ethyl methyl carbonate (EMC), and diethyl carbonate (DEC) at volume ratio of 4:2:4. Cyclic voltammetry (CV) and electrochemical impedance spectroscopy (EIS) were measured by electrochemical workstation (CHI760E). The galvanostatic charged and discharged characteristics were tested in a voltage range from 0.01 to 3.0 V with LAND CT2001A battery tester.

## Results and Discussion

**Fig. 1:**
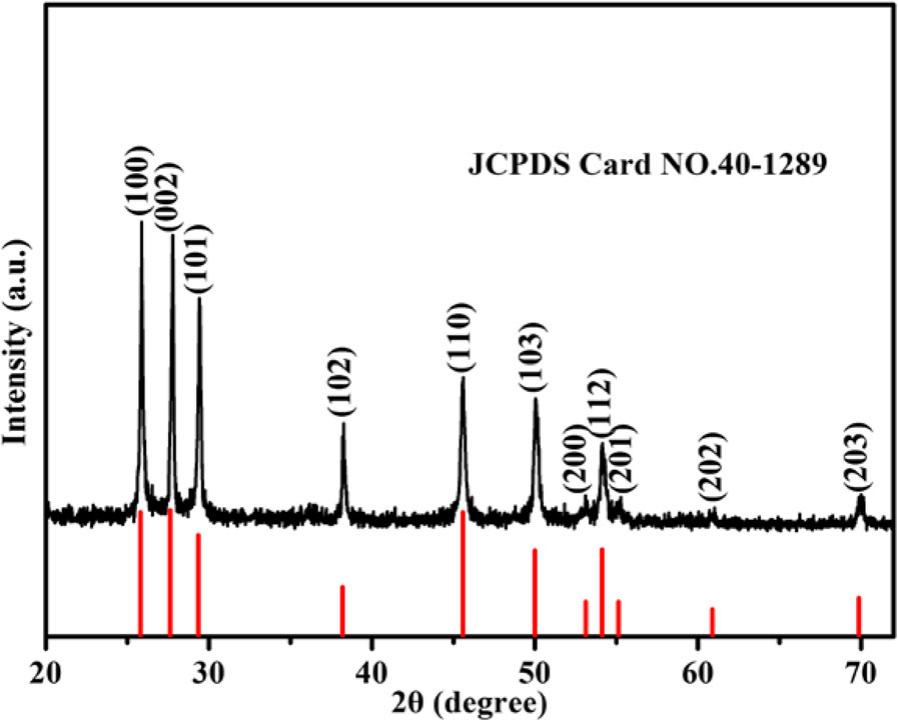
XRD pattern of the as-obtained *γ*-MnS microstructures

The phase purity of the as-prepared product was investigated using the power X-ray diffraction (XRD) and energy-dispersive spectrometer (EDS). Figure 1 is the XRD pattern of the as-synthesized sample, on which all the diffraction peaks can be indexed to be the hexagonal phased *γ*-MnS. The lattice constants of the as-synthesized sample are calculated to be *a* = *b* = 3.9792 Å, *c* = 6.4469 Å, *α* = *β* = 90.0°, and *γ* = 120.0°, which is consistent with the literature value (JCPDS card no. 40-1289). No other peak is detected, indicating high purity of the as-prepared sample.

**Fig. 2:**
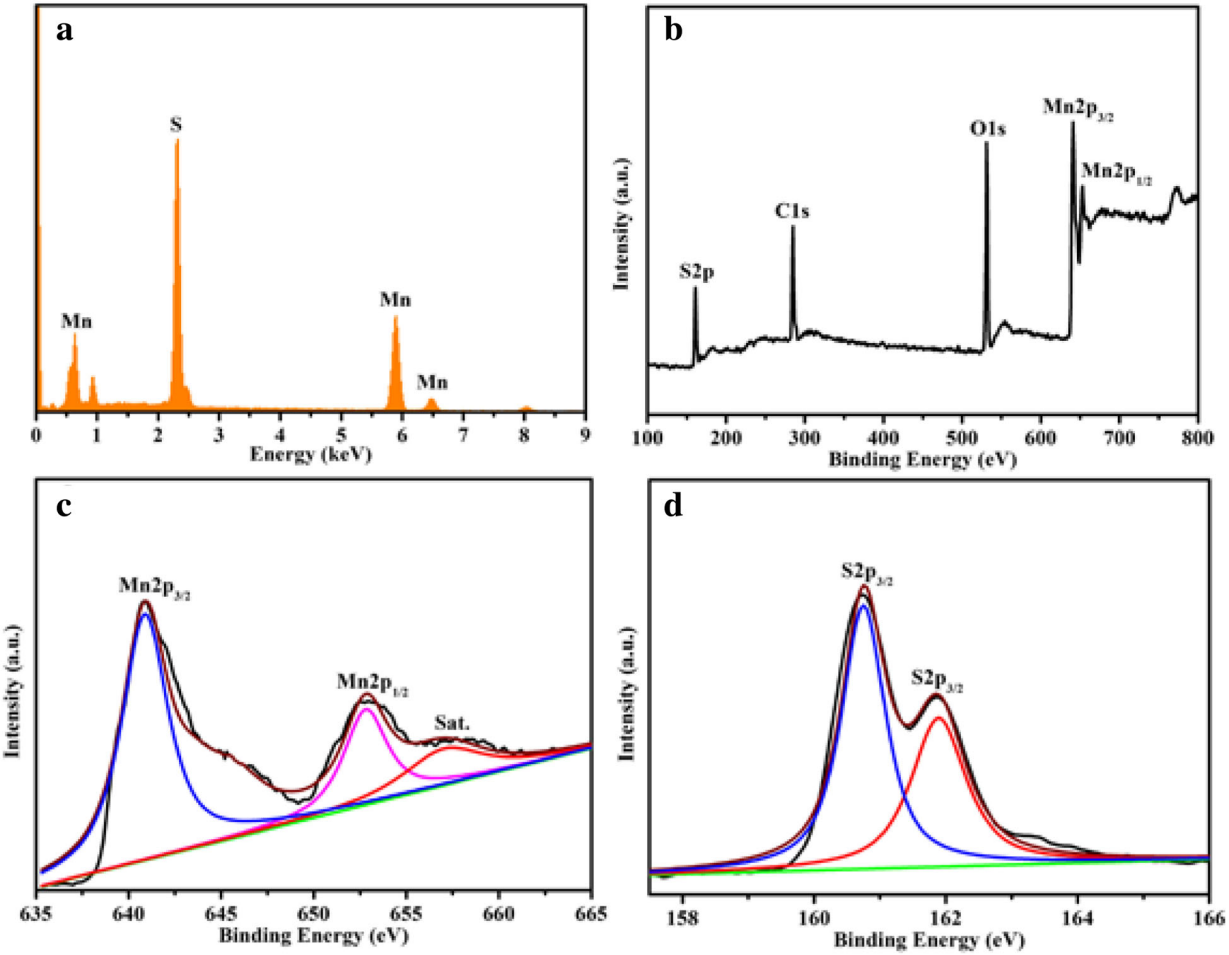
**a** EDS spectrum of the as-obtained *γ*-MnS, **b** overall survey, **c** Mn 2p, and **d** S 2p XPS spectra of the as-prepared *γ*-MnS microstructures

The chemical composition of the as-prepared sample is further investigated using X-ray energy-dispersive spectroscopy (EDS), and the corresponding result is shown in Fig. 2a. The corresponding result clearly confirms the existence of elements Mn and S. The atom ratio between Mn and S is determined to be 0.49:0.51, which is in accordance with the theoretical value of MnS. The XPS spectra of the as-synthesized *γ*-MnS are shown as Fig. 2b–d. The overall XPS spectrum of the as-synthesized *γ*-MnS clearly indicates the existence of elements Mn and S (Fig. 2b). The Mn 2p spectrum (Fig. 2c) consists of two peaks with binding energies centering at ~640.8 and ~652.8 eV, which are the characteristic peaks of Mn 2p_3/2_ and Mn 2p_1/2_, respectively [22]. Similarly, the S 2p spectrum (Fig. 2d) was also composed of two peaks with binding energies of ~160.7 and ~161.9 eV, corresponding to S 2p_3/2_ [23]. The XPS results are in well agreement with the XRD and EDS analysis, indicating that the high purity of the as-obtained *γ*-MnS microstructures.

**Fig. 3:**
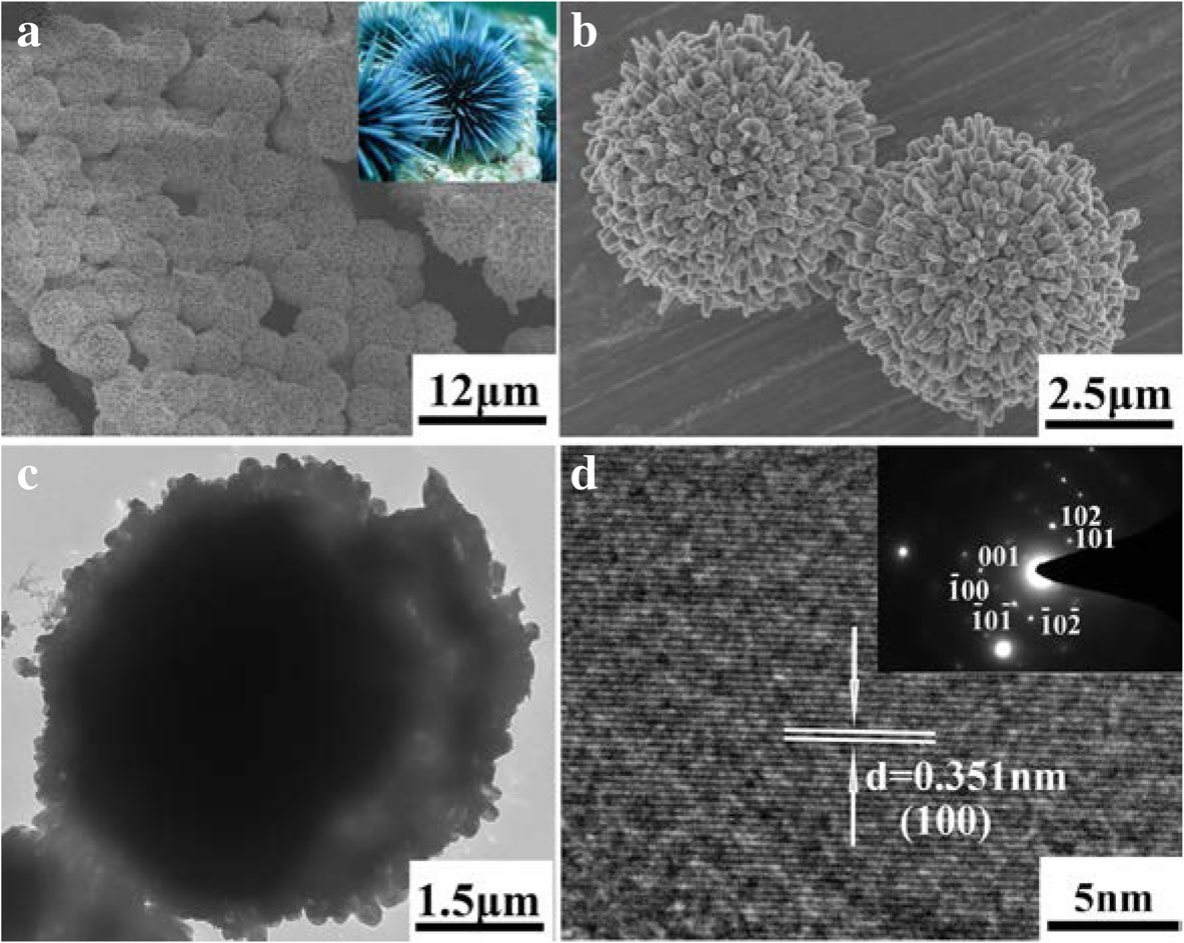
**a**, **b** Low- and high-magnification FESEM image of the as-prepared *γ*-MnS microstructures, **c** TEM image, and **d** HRTEM image of the as-prepared *γ*-MnS microstructures (*inset* of image **d** - SAED pattern of *γ*-MnS microstructures)

The morphology and microstructure of the as-synthesized *γ*-MnS nanostructures were further investigated using FESEM, TEM, and HRTEM. Figure 3a is the low-magnification SEM image of the as-obtained *γ*-MnS microstructures, which clearly indicate that the as-obtained sample is mainly composed of urchin-like structures. The high-magnification SEM image of the as-prepared sample further indicates that the as-obtained urchin-like structure has a diameter of ca. 5 μm with acicular crystallites radiating from the center with a uniform size distribution (Fig. 3b). The nanorods grown from the center have uniform diameters of about 150 nm and lengths up to 700 nm. Figure 3c is the TEM image of a single *γ*-MnS nanostructure, which clearly confirms the urchin-like structure of the as-prepared *γ*-MnS. To get further insight into the detailed structure of the as-synthesized *γ*-MnS, SAED and HRTEM were employed. The inset of Fig. 3d is the SAED pattern of *γ*-MnS, which clearly indicates the single-crystallized nature of the nanorods. Figure 3d is the HRTEM image of a single nanorod from the urchin-like nanostructure, in which clear lattice fringes can be observed. The typical lattice spacing, being determined to be 0.351 nm, corresponds to the (100) lattice plane of hexagonal phased *γ*-MnS.

**Fig. 4:**
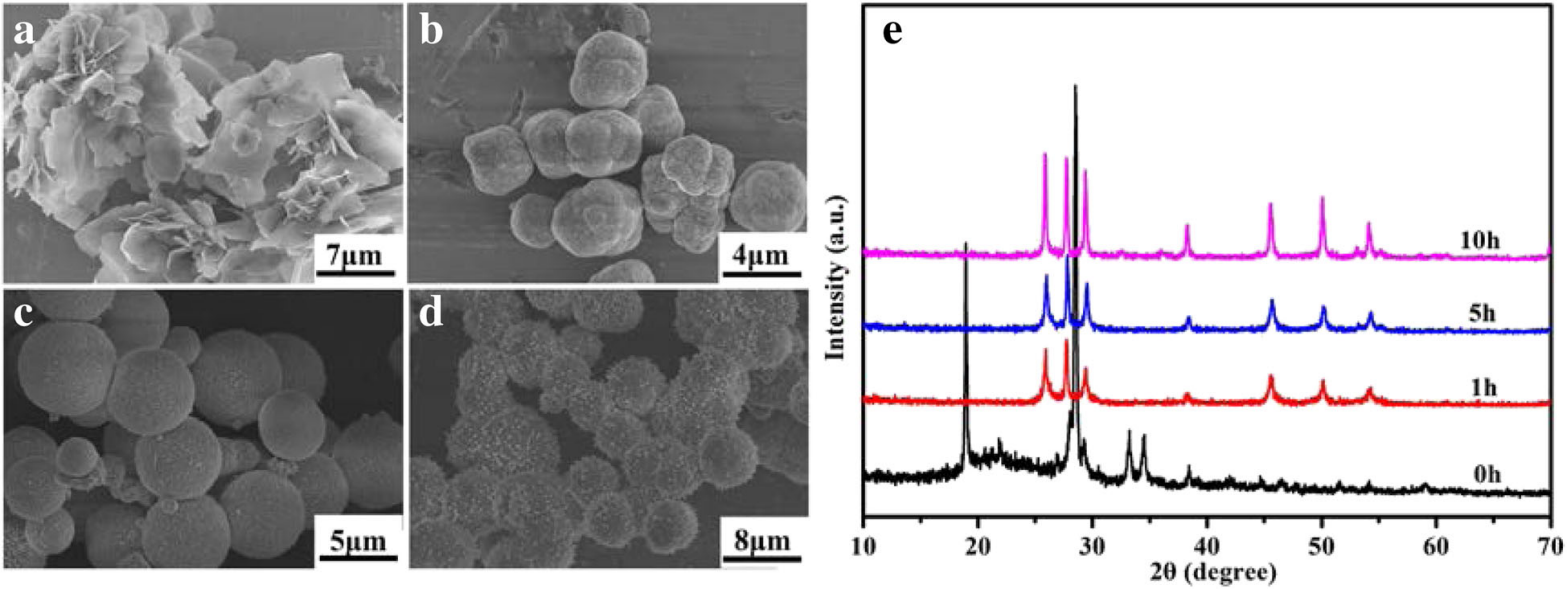
FESEM images of the intermediate products obtained at different reaction stages: **a** 0 h, **b** 1 h, **c** 5 h, and **d** 10 h. **e** XRD patterns of the intermediate products obtained at different reaction stages

To understand the growth mechanism of the urchin-like *γ*-MnS microstructures, experiments that involved the intermediate products at different reaction times were carried out. Figure 4a is the SEM image of the product obtained after heating at 70 °C for 2 h, which clearly indicates that the sample is composed of a large number of nanoplates. According to the XRD result, these nanoplates can be designated to be the complex that forms between the l-cysteine and Mn^2+^. As the reaction had been carried out at 180 °C for 1 h, these nanoplates gradually vanished and a large number of irregular microparticles appeared (Fig. 4b). The as-formed irregular microparticles are determined to be *γ*-MnS, indicating the transformation from Mn-l-cysteine complex to *γ*-MnS. In the next step, these irregular microparticles gradually transform to *γ*-MnS microspheres with diameters of about 5 μm (Fig. 4c). A careful observation of these microspheres further indicates that the surfaces of these microspheres are not smooth. A large number of nanoparticles with diameters of about 30 nm can be clearly observed on the surfaces of these microspheres, which will serve as the nuclei centers for the growth of *γ*-MnS nanorods in the next step. As the reaction went on further, these small nanoparticles will gradually transform to small nanorods. After reacting for 18 h, the urchin-like nanostructure would finally form.

**Scheme 1:**
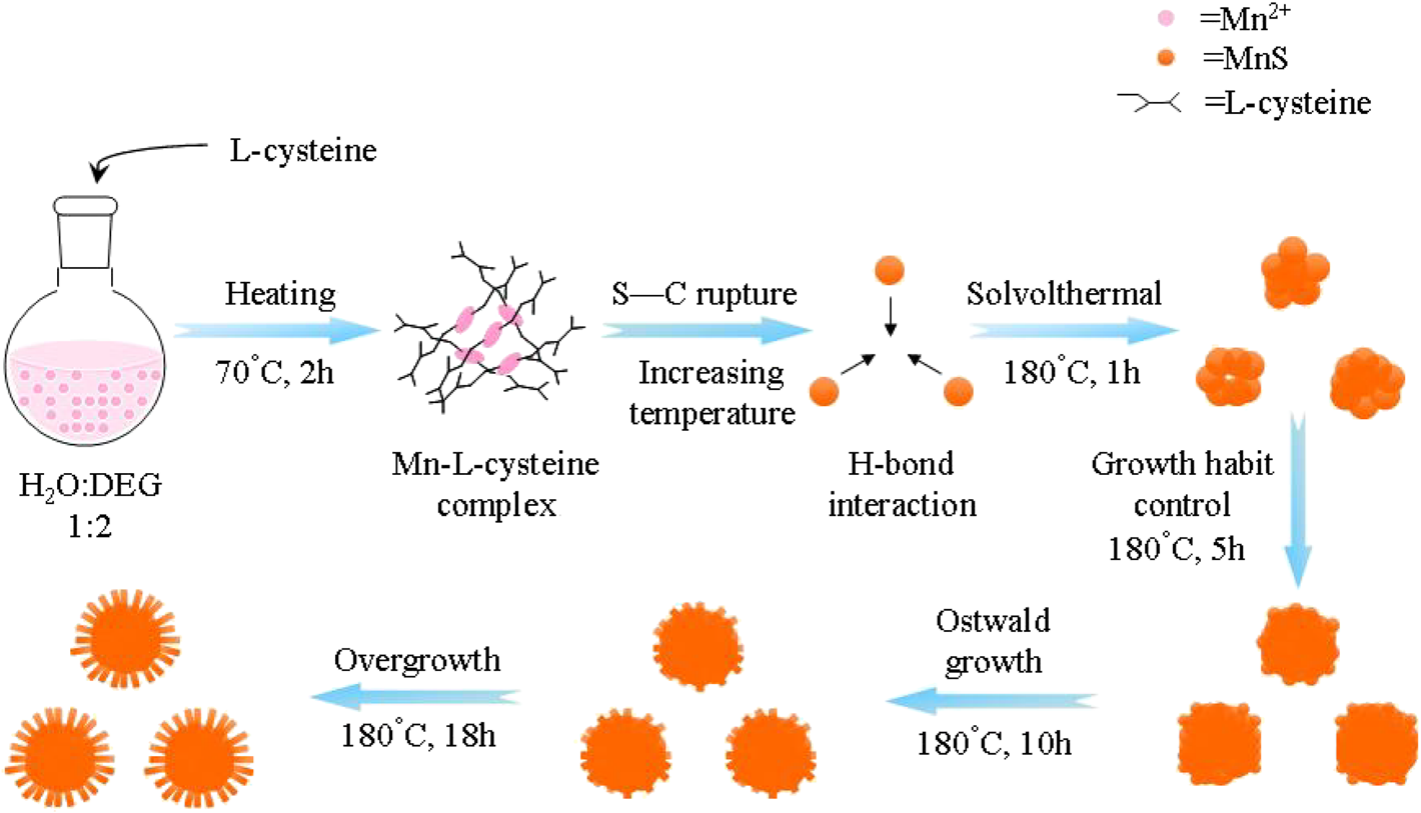
Schematic illustration for the formation process of the urchin-like *γ*-MnS microstructures

Based on the previous reports and the time-dependent experiments, a two-step formation mechanism for the urchin-like *γ*-MnS microstructures is proposed (Scheme 1). Owing to the functional groups in the molecules (–SH, –NH_2_, and –COOH), l-cysteine molecule is commonly employed for the preparation of metal sulfides with different morphologies [7, 24]. These functional groups usually lead to the versatile role of l-cysteine during the synthetic process, such as sulfur sources, polydentate ligands, self-assembly reagent, or even shape-controlling agent [25–32]. When Mn^2+^ and l-cysteine were mixed in the solvent, a complex between Mn^2+^ and l-cysteine will form, leading to the formation of nanoplates at the initial stage. When the solvothermal reaction began, the initial formed Mn complex will decompose, leading to the formation *γ*-MnS microparticles. The whole chemical process can be described as

$$ {\mathrm{Mn}}^{2+}+\mathrm{n}\ \mathrm{L}\hbox{-} \mathrm{cysteine}\overset{\mathrm{coordination}}{\to }{\left[\mathrm{M}\mathrm{n}{\left(\mathrm{L}\hbox{-} \mathrm{cysteine}\right)}_{\mathrm{n}}\right]}^{2+} $$

$$ {\left[\mathrm{M}\mathrm{n}{\left(\mathrm{L}\hbox{-} \mathrm{cysteine}\right)}_{\mathrm{n}}\right]}^{2+}\overset{\mathrm{Mn}\hbox{-} \mathrm{S}\ \mathrm{rupture}\ }{\to}\mathrm{M}\mathrm{n}\mathrm{S} $$

In our synthesis, l-cysteine is excessive. The free thiol groups of the excessive cysteine molecule will bind to the surfaces of the initial formed *γ*-MnS nanoparticles. Meanwhile, the hydrogen bonds and S-S bonds will form between the cysteine molecules. Driven by the interactions of hydrogen bonds among tiny particles, H_2_O and DEG, the as-formed *γ*-MnS tiny nanoparticles will cross-link together, leading to the formation of irregular microparticles when the solvothermal reaction has been conducted for 1 h [7]. As the solvothermal reaction has been conducted at 180 °C for 5 h, these irregular aggregates will gradually transform to *γ*-MnS microspheres via the Ostwald ripening process [8, 24, 33–36]. Because of the strong interactions between cysteine and Mn^2+^, the growth rate of *γ*-MnS can be effectively controlled. The growth rate in all the direction is nearly the same under the influence of l-cysteine, leading to the formation of *γ*-MnS microspheres in this step. As the reaction went on, the influence of l-cysteine gradually weakens because of the thermal decomposition process during the solvothermal process. Driven by the intrinsic anisotropic growth habit of *γ*-MnS, the 1D *γ*-MnS nanorods will gradually form on the surfaces of the *γ*-MnS microspheres. As the solvothermal reaction went on, the as-formed nanorods grow longer and longer. When the reaction has been conducted for 18 h, the urchin-like microstructures finally form.

**Fig. 5:**
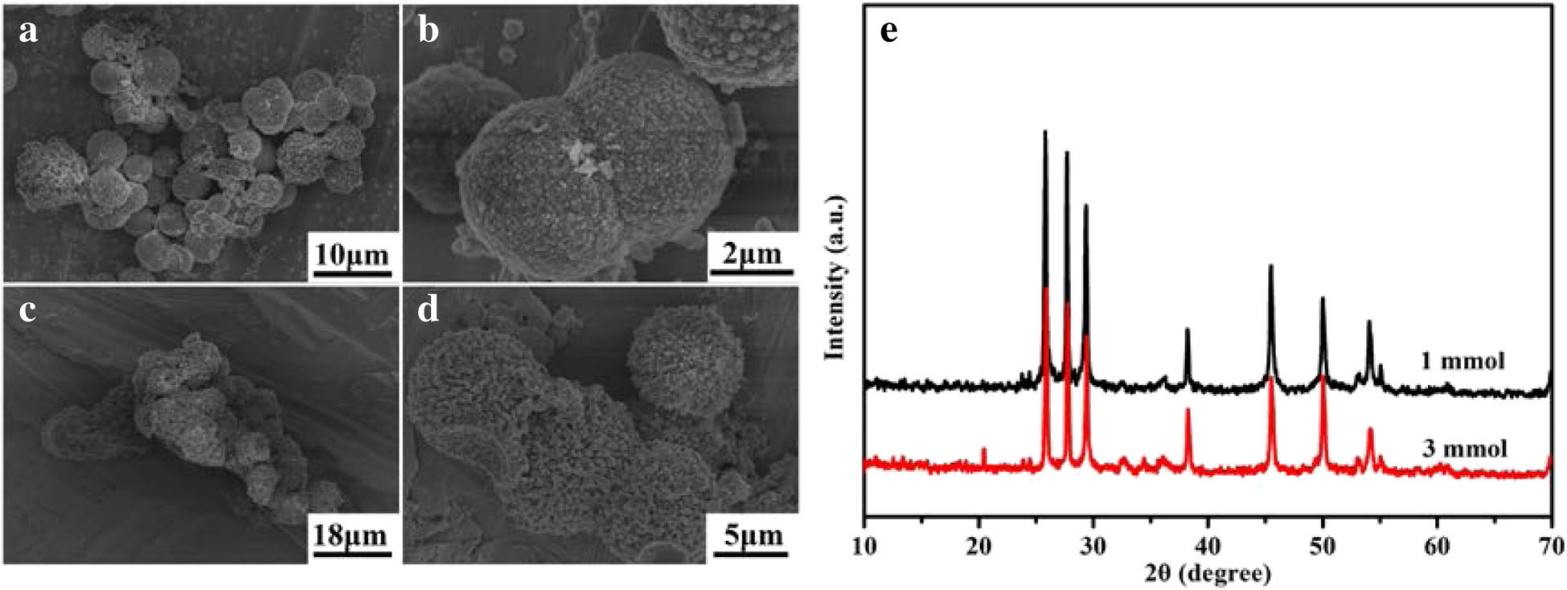
**a**, **b** Low- and high-magnification FESEM images of as-prepared *γ*-MnS when 1 mmol l-cysteine was used. **c**, **d** Low- and high-magnification FESEM images of as-prepared *γ*-MnS when 3 mmol l-cysteine was used. **e** XRD patterns of as-prepared *γ*-MnS when a different amount of l-cysteine was employed

To further investigate the effect of l-cysteine during the synthetic process, comparative experiments employing different amount of l-cysteine were carried out. According to the XRD patterns of the samples when different amount of l-cysteine was employed, the final products under all conditions can be designated to be the hexagonal phased *γ*-MnS (JCPDS card no. 40-1289). Figure 5a, b are the low- and high-magnification FESEM images of the as-prepared *γ*-MnS microstructure when 1 mmol l-cysteine was employed, which clearly indicate that the sample is composed of irregular microspheres. Careful observation further indicates that there are some small nanorods on the surfaces of these microspheres. When the amount of l-cysteine increased to 3 mmol, the products are mainly composed of some urchin-like structures as well as some irregular microparticles. As we have mentioned above, l-cysteine not only acts as the sulfur source but also acts as a morphological-controlling agent during the synthetic process. The formation of the complex between Mn and l-cysteine exerts great influence on the formation rate of MnS, which is of vital importance for the morphological control of the final product. When the concentration of l-cysteine is low, the reaction speed is high, which will result in the formation of the irregular microspheres. When the concentration of l-cysteine is high, the reaction rate becomes low, which would be beneficial for the morphological control of the final product. However, the low reaction rate will also inevitably lead to the incomplete transformation from the irregular microparticles to the urchin-like structures. And this can be used to explain the phenomenon why irregular microparticles as well as urchin-like structures co-exist in the obtained sample when 3 mmol l-cysteine was employed.

**Fig. 6:**
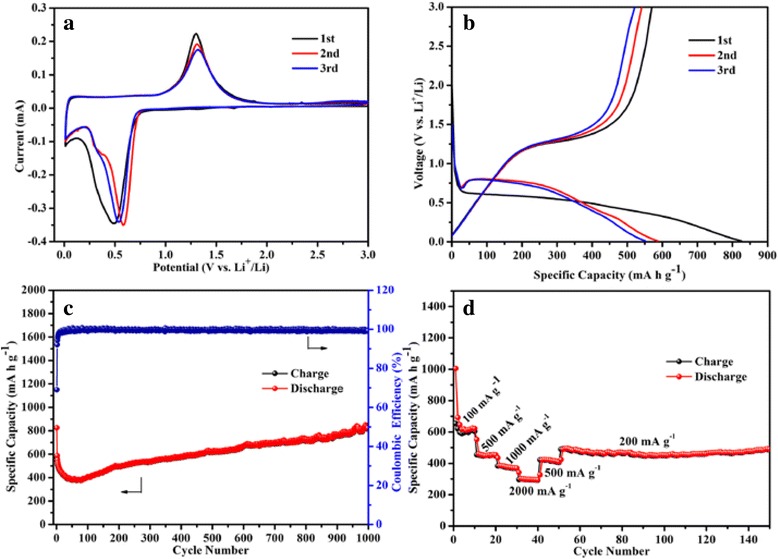
**a** CV curves of electrode containing *γ*-MnS in the voltage range of 0 to 3.0 V, **b** the voltage profiles between 0.01 and 3.00 V at the current density of 500 mA g^−1^, **c** cycling performance of *γ*-MnS electrode at current density of 500 mA g^−1^, and **d** rate performance of electrode containing *γ*-MnS at different current densities from 100 to 2000 mA g^−1^

As it is shown in Fig. 6, the as-prepared urchin-like *γ*-MnS exhibits excellent electrochemical performance. Figure 6a shows the cyclic voltammetry (CV) curves between 0 to 3.0 V at a scan rate of 0.1 mV s^−1^. Two peaks can be observed during the first cycles. The reduction peak centering at 0.49 V can be assigned to the reduction of Mn^2+^ to metallic Mn, while the oxidation peak centering at 1.3 V corresponds to the insertion of Li^+^ into the *γ*-MnS lattice to form a homogeneous phase of Li_2_MnS [18, 20, 37, 38]. From the second cycle, the reduction peak shifts from 0.49 to 0.54 V, which could be ascribed to the formation of solid electrolyte interphase (SEI) layer and lithium-driven structural modifications [39–41]. The electrochemical reaction can be summarized by the following equation:

1 $$ \mathrm{M}\mathrm{n}\mathrm{S}+2\mathrm{L}{\mathrm{i}}^{+}+2{\mathrm{e}}^{-}\to \mathrm{L}{\mathrm{i}}_2\mathrm{M}\mathrm{n}\mathrm{S} $$

2 $$ \mathrm{L}{\mathrm{i}}_2\mathrm{M}\mathrm{n}\mathrm{S}+2{\mathrm{e}}^{-}\to \mathrm{M}\mathrm{n}+\mathrm{L}{\mathrm{i}}_2\mathrm{S} $$

Figure 6b shows the discharge/charge voltage profiles of the *γ*-MnS of different cycles at the current of 500 mA g^−1^ between 0.01 and 3.0 V. According to the discharge/charge profiles, the initial discharge capacity is 825.5 mA h g^−1^ and the corresponding columbic efficiency is about 69.06 %. The loss in initial columbic efficiency can be attributed to be the formation of the SEI layer and decomposition of the electrolyte, which have been widely reported for many anode materials [42–45]. The discharge and charge plateaus of the as-prepared *γ*-MnS electrode are ~0.65 and ~1.25 V, respectively, which are in well agreement with the CV results. The discharging voltage plateau in the second cycle is much higher than the corresponding value in the first cycle, indicating the irreversible reactions of *γ*-MnS and formation of unstable SEI layer [17, 38, 46, 47]. More interesting, in the third cycle, the discharge and charge plateaus became stable, which is in accord with the cycling performance as shown in Fig. 6c.

**Fig. 7:**
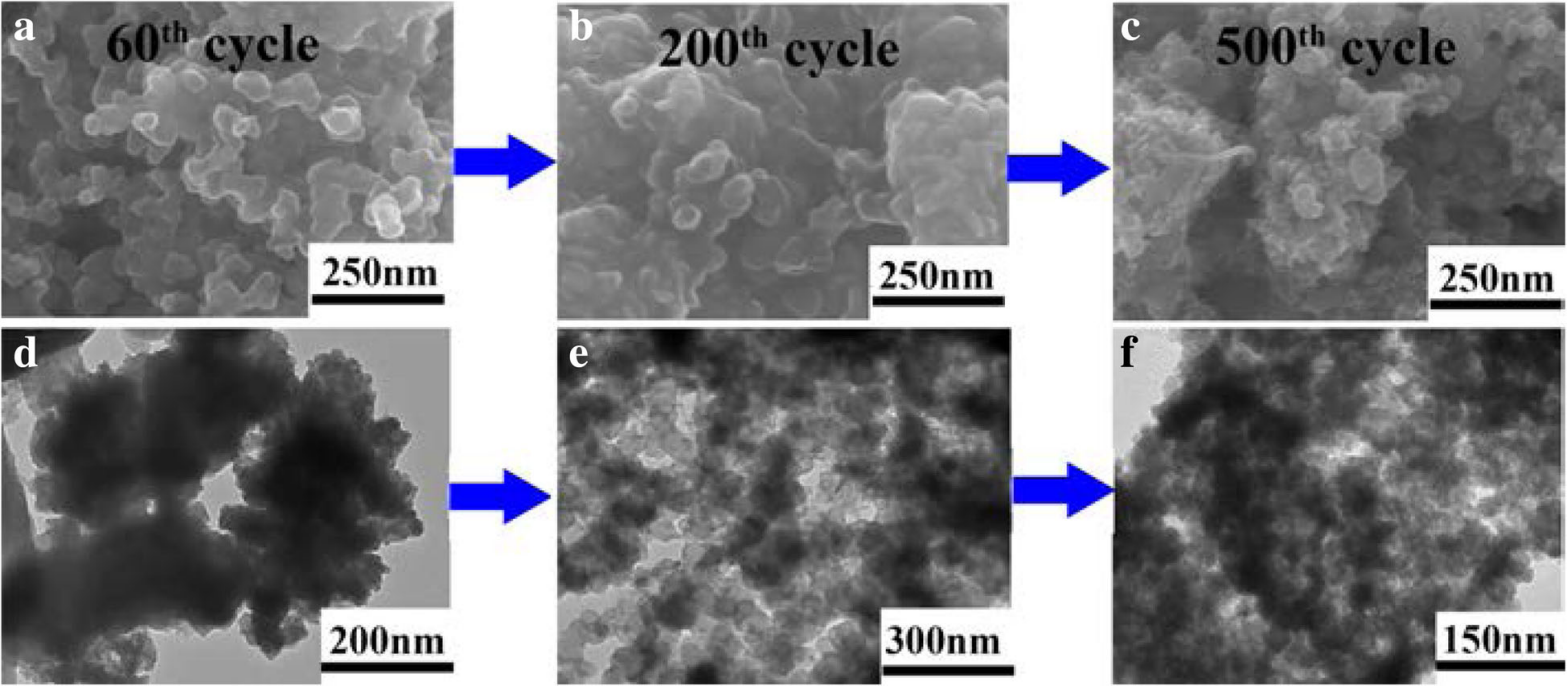
SEM image evolution of *γ*-MnS after charge/discharge for **a** 60th, **b** 200th, and **c** 500th cycles. TEM images of *γ*-MnS after charge/discharge for **d** 60th, **e** 200th, and **f** 500th cycles

**Fig. 8:**
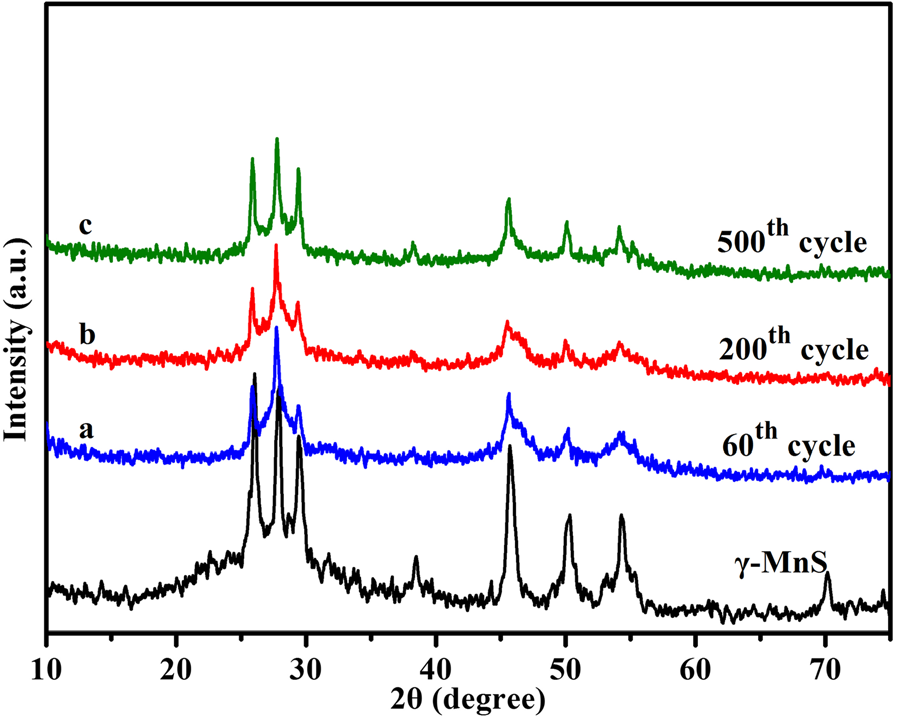
XRD patterns of *γ*-MnS after charging/discharging for **a** 60, **b** 200, and **c** 500 cycles

Figure 6c presents the cycling performance of the as-prepared *γ*-MnS at current density of 500 mA g^−1^. An obvious decrease in the discharge capacity can be clearly observed in the initial 60 cycles. The discharge capacity of the as-prepared *γ*-MnS fades significantly in the following cycles, decreasing from 587.8 mA h g^−1^ at second cycle to 379.6 mA h g^−1^ at 60th cycle. This phenomenon could be attributed to the lithiation-induced mechanical degradation and the formation of the unstable SEI layer [47, 48]. To illustrate this view, the coin cell after 60th cycles is disassembled and investigated using FESEM (Fig. 7) and XRD (Fig. 8). According to the XRD pattern, the phase of the active material remains unchanged after the charge/discharge process. However, the urchin-like microstructures disappears and transforms to irregular aggregates. Thus, the pulverization of the active material can be regarded as the main reason for the discharge capacity fading in the initial 60 cycles. After charge/discharge for 60 cycles, the discharge capacity gradually increases. To be specific, the discharge capacity increases from 378 mA h g^−1^ at the 60th cycle to 823.4 mA h g^−1^ in the 1000th cycle. It is interesting to note that the discharge capacity after 1000 cycles is higher than the theoretical value of *γ*-MnS, which may result from the so-called “pseudo-capacitance behavior.” This phenomenon is attributed to the reactivated process and stable SEI layer optimization [49, 50]. To get further insight into the capacity decrease in the cycling process, the coin cells after 200th and 500th cycles are also disassembled and investigated using XRD, SEM, and TEM. XRD patterns of the samples clearly indicate that the phase of the active material remains unchanged during the whole charge/discharge process. However, the SEM and TEM observations of the active material clearly indicate the size changes during the charge/discharge process. Upon the increase of the charge/discharge cycles, the active material gradually transforms from irregular microparticles to small nanoparticles. And the size of the active material decreases to 30 and 20 nm upon the 200th and 500th cycle. The size decrease of the active material will provide more active sites for the storage of lithium ions, which is beneficial for the maintenance of electrochemical performance during long cycle. Meanwhile, the irreversible electrochemical reaction during the charge/discharge process will lead to the formation of metallic Mn during the charge/discharge process. The formation of metallic Mn will increase the electrical conductivity of the electrode, which will greatly facilitate the insertion/extraction of lithium ions and transport of electrons, leading to the increment of discharge capacity in the following cycles [43]. What is more, the as-formed metallic Mn could also act as a catalyst for the decomposition of the SEI layer and the reversible formation/dissolution of the organic polymeric film, which would lead to the reversible increase in the discharge capacity through the so-called “pseudo-capacitance behavior.” The anode material is keeping hundreds of cycles without capacity fading, indicating that the mechanical degradation can effectively restructure the active material and optimize SEI layer [47].

Figure 6d presents the rate performance of the urchin-like *γ*-MnS in LIB applications. Herein, the as-prepared *γ*-MnS was tested under different charge current densities from 100 to 2000 mA g^−1^. When the current density is 100 mA g^−1^, the reversible capacity keeps at ~620 mA h g^−1^ after 10 cycles. When the current density increased to 500, 1000, and 2000 mA g^−1^, the corresponding reversible capacities are 450, 370, and 300 mA h g^−1^, respectively. Obviously, the *γ*-MnS anode material shows excellent performance at a different current density. Even being cycled at 2000 mA g^−1^, the capacity can still reach 300 mA h g^−1^. When the current density returns to 200 mA g^−1^, the capacity of *γ*-MnS can reach to 490 mA h g^−1^, which is much higher than that of graphite in the same condition [51, 52]. The rate performance is in accord with the discharge/charge voltage profiles and the cycling performance. A comparison between the reported MnS-based anode material [12, 16, 18, 19] and our product is summarized in Table 1. Obviously, the as-synthesized *γ*-MnS in this work shows good electrochemical performance.

**Table 1:** Comparison of electrochemical performance with previously reported structure

Material	Current density (mA g^−1^)	Reversible capacity (mA h g^−1^)/cycles	Reference
Urchin-like *γ*-MnS	500	823.4/1000th	This work
MnS nanoparticles	C/5	470/100th	[12]
*γ*-MnS/rGO	200	600/100th	[16]
Hollow micro-sphere MnS/RGO	500	830/100th	[17]
Coral-like α-MnS@NC	500	699/400th	[18]
MnS-C	500	636/100th	[19]

**Fig. 9:**
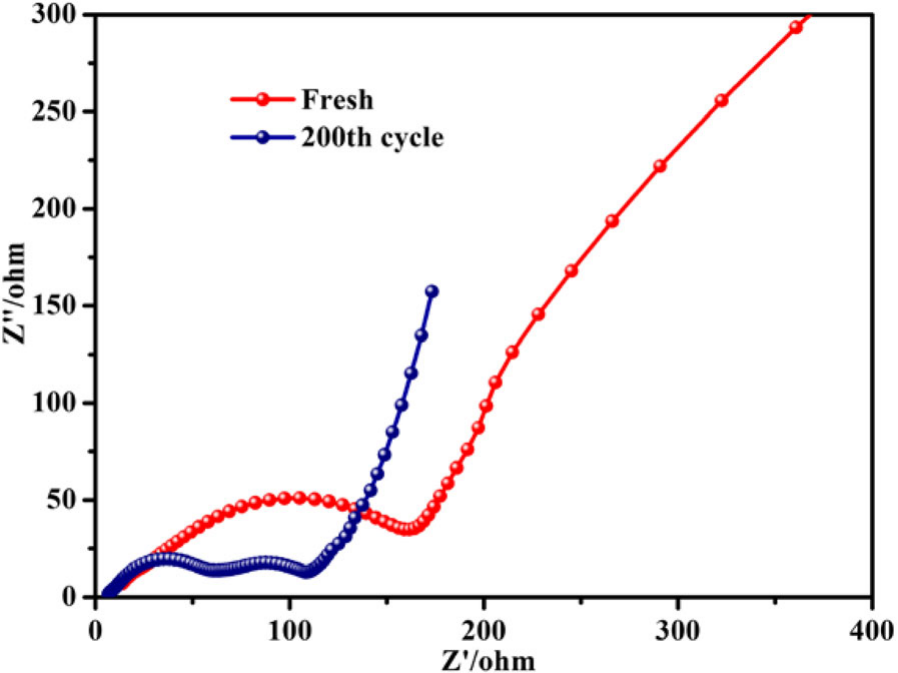
EIS curves of sphere MnS before discharging and after 100 cycles of charging/discharging at a current density of 500 mA g^−1^ in the frequency range from 0.01 Hz to 100 kHz

To further understand the electrochemical properties of the urchin-like *γ*-MnS, electrochemical impedance spectroscopy (EIS) was measured at frequencies from 0.01 Hz to 100 kHz and the result is shown as Fig. 9. Nyquist impedance plots indicate that the diameter of the semicircle for sphere-like *γ*-MnS without charge/discharge process is larger than the corresponding value after 200 cycles, which clearly indicates the formation of the lithium channels and the degradation of the anode material, which is well in accordance with SEM and TEM images at 200th cycle as shown in Fig. 7b, e. In the medium- and high-frequency regions, the single semicircle before the cycles turns into two after 200 cycles, indicating the formation of SEI film.

## Conclusions

In summary, urchin-like *γ*-MnS structures were successfully synthesized by a facile solvothermal method using l-cysteine and MnCl_2_ · 4H_2_O as the raw materials. A two-step growth mechanism has been proposed for the formation of the urchin-like *γ*-MnS structures. The product exhibits excellent cycling stability (823.4 mA h g^−1^ at the current density of 500 mA g^−1^ after 1000 cycles) and the discharge voltage is ~0.75 V. SEM, TEM, and XRD were employed to investigate the transformation of the active materials during the charge/discharge process, which clearly indicate that the structural degradation and reformation of the active material play the key roles for the maintenance of the cycling stability. Considering the simple preparation process, excellent cycling stability, and low voltage potential of the as-prepared sample, the product is considered to be a potential candidate anode material in LIBs.
